# Draft Genome Sequence of *Microvirga* sp. Strain BSC39, Isolated from Biological Soil Crust of Moab, Utah

**DOI:** 10.1128/genomeA.01197-14

**Published:** 2014-11-13

**Authors:** Alexis C. Bailey, Matthew Kellom, Amisha T. Poret-Peterson, Kathryn Noonan, Hilairy E. Hartnett, Jason Raymond

**Affiliations:** a School of Earth and Space Exploration, Arizona State University, Tempe, Arizona, USA; b Department of Chemistry and Biochemistry, Arizona State University, Tempe, Arizona, USA

## Abstract

*Microvirga* sp. BSC39 was isolated from a biological soil crust near Moab, Utah. The strain appears to be capable of chemotaxis and exopolysaccharide synthesis for biofilm adhesion. The BSC39 genome contains iron siderophore uptake and hydrolysis enzymes; however, it lacks siderophore synthesis pathways, suggesting the uptake of siderophores produced by neighboring microbes.

## GENOME ANNOUNCEMENT

*Microvirga* sp. strain BSC39 was collected in an environmental sample from the Green Butte site near Moab, Utah (N38°42′56.2″, W109°41′32″) in May 2009 from a biological soil crust (BSC) (1). BSCs are microbial communities that form in arid and semiarid environments and supply bioavailable carbon (2), nitrogen (3), and metal-containing compounds (4) to their surroundings via carbon and nitrogen fixation as well as siderophore synthesis (5; K. Noonan, A. T. Poret-Peterson, A. D. Potrafka, A. D. Anbar, F. Garcia-Pichel, and H. E. Hartnett, unpublished data). Here, we present the draft genome of *Microvirga* sp. BSC39, a heterotrophic, siderophore-hydrolyzing member of *Alphaproteobacteria*.

BSC39 was cultured and isolated on BG-11 agar plates and modified to detect the production of siderophores (6). DNA was extracted from the isolate using the Ultra-Clean Soil DNA Extraction kit, prepped for sequencing using the Illumina TruSeq DNA HT sample prep kit, and sequenced on 21 June 2014 at the Genomics Core of The Biodesign Institute at Arizona State University using the Illumina MiSeq (Illumina RTA 1/18/54). A total of 833,789 300-bp paired-end reads were generated, resulting in 335.1 Mb of raw sequence data. We assembled 18,135 Rho *N*_50_ unitigs averaging 419 bp in length and remaining reads into 80 gapless scaffolds using the Celera Whole-Genome Shotgun Assembler version 8.1 for a total genome length of 5,655,736 bp (50× coverage) (7). The scaffolds were screened for DNA contaminants and annotated by the NCBI Prokaryotic Genome Annotation Pipeline, which identified 4,793 genes (8). 16S ribosomal analysis showed 99% identity to the 16S sequence of *Microvirga aerilata* from the NCBI 16S Ribosomal RNA Sequences Database using BLAST (9). The G+C content was 62.94%, which is consistent with the *Microvirga* genus (10).

Based on the genome of BSC39, it appears to be an opportunistic heterotrophic member of the soil crust microbial community with a complete TCA cycle and terminal cytochrome oxidases, metabolizing carbon and also nitrogen biomolecules produced by other BSC microbes. BSC39 has genes for chemotaxis, flagellar motility, and exopolysaccharide biofilm synthesis, which facilitate movement toward and adhesion to areas of favorable nutrient conditions. BSC39 also appears to be capable of iron acquisition via siderophore uptake by iron siderophore ABC transporter permease and siderophore hydrolysis. The genes producing siderophores do not appear to be present within the BSC39 genome, and therefore any siderophores taken up and hydrolyzed for iron acquisition must have been produced by other microbes within the microbial community.

### Nucleotide sequence accession numbers.

This whole-genome shotgun project has been deposited at DDBJ/EMBL/GenBank under accession number JPUG00000000. The version described in this paper is version JPUG01000000.
